# Macro- and Microstructural Alterations in the Midbrain in Early Psychosis Associates with Clinical Symptom Scores

**DOI:** 10.1523/ENEURO.0361-24.2025

**Published:** 2025-03-17

**Authors:** Zicong Zhou, Kylie Jones, Elena I. Ivleva, Luis Colon-Perez

**Affiliations:** ^1^Department of Pharmacology and Neuroscience, University of North Texas Health Science Center, Fort Worth, Texas 76107; ^2^Department of Psychiatry, University of Texas Southwestern Medical Center, Dallas, Texas 75390

**Keywords:** diffusion MRI, early psychosis, Human Connectome Project, midbrain, structural MRI

## Abstract

Early psychosis (EP) is a critical period for psychotic disorders during which the brain undergoes rapid and significant functional and structural changes ( Shinn et al., 2017). The Human Connectome Project (HCP) is a global effort to map the human brain's connectivity in health and disease. Here we focus on HCP-EP subjects (i.e., those within 5 years of the initial psychotic episode) to determine macro- and microstructural alterations in EP (HCP-EP sample, *n* = 179: EP, *n* = 123, controls, *n* = 56) and their association with clinical outcomes (i.e., symptoms severity) in HCP-EP. We carried out analyses of deformation-based morphometry (DBM), scalar indices from the diffusion tensor imaging (DTI), and tract-based spatial statistics (TBSS). Lastly, we conducted correlation analyses focused on the midbrain (DBM and DTI) to examine associations between its structure and clinical symptoms. Our results show that the midbrain displays robust alteration in its structure (DBM and DTI) in the voxel-based analysis. Complimentary alterations were also observed for the hippocampus and putamen. A seed-based analysis centered around the midbrain confirms the voxel-based analysis of DBM and DTI. TBSS displays structural differences within the midbrain and complementary alterations in the corticospinal tract and cingulum. Correlations between the midbrain structures and behavior showed that the quantified features correlate with cognition and clinical scores. Our findings contribute to understanding the midbrain-focused circuitry involvement in EP and provide a path for future investigations to inform specific brain-based biomarkers of EP.

## Significance Statement

Psychotic disorders are preceded by a critical period known as early psychosis (EP), where detection and effective interventions could substantially improve symptoms and have the potential to modify the subsequent disease course. However, early biomarkers of the disease are not established, and the current clinical MRI practices do not contribute to diagnosis or treatment. Therefore, identifying critical and comprehensive features of brain alteration (e.g., via multimodal MRI approaches) for EP is a high priority for the field to determine future impactful biomarkers. The HCP-EP provides a refined, high-quality, and specific dataset focusing on the EP period (i.e., those within 5 years of the initial psychotic episode), which we focus on to provide insights into putative early targets of EP.

## Introduction

Early psychosis (EP) represents a critical period in psychotic disorders (Shinn et al., 2017) when early detection and comprehensive interventions can substantially improve symptom severity and potentially modify subsequent disease course (Kane et al., 2016; Xiao et al., 2018; Blessing et al., 2020). While the clinical manifestations of EP have been extensively characterized (Hardy et al., 2019), the development of objective, measurable biomarkers that can augment clinical diagnoses and inform actionable targets for interventions remains limited. A meta-analysis suggests that ∼6% of individuals presenting with first-episode psychosis demonstrate clinically relevant findings on MRI (Blackman et al., 2023). Clinical findings refer to the overt presentation of lesions or observable radiological outcomes in clinical MR images. The sparse number of positive clinical findings highlights the need for better and objective MRI biomarkers in EP. Psychotic disorders’ heterogeneity of etiology coupled with distinct effects in the brain suggest that a single biomarker will not capitulate the critical features of EP; hence, a “biomarker profile” may be the final goal (Mirzakhanian et al., 2014). Even though several discrete biomarkers have already been suggested as candidates, there is an outstanding need for comprehensive, in-depth, in vivo brain characterization with robust cognitive, symptom, and functional benchmarks to lead to a “biomarker profile” and inform novel individualized precision treatments (Ji et al., 2021).

A recent focus in the biomarker search of psychotic disorders is determining network alterations of the brain's structure and function (Rubinov and Bullmore, 2013; Braun et al., 2016; O'Neill et al., 2019). Network modeling studies show that white matter (i.e., network edges) serves as a conduit of pathology in psychotic disorders, which exacerbates pathology in the advanced stage of the disease (Chopra et al., 2023). The anterior hippocampus is considered a significant network hub of early functional alterations (Schobel et al., 2013) and serves as a relay for the proliferation of pathology into the posterior and prefrontal cortex (Chopra et al., 2023). Independently, the frontal cortex has also been shown to display reductions in cortical thickness in clinical high-risk individuals who subsequently convert to psychosis (Cannon et al., 2015). The cortical and hippocampal focus has been validated in a recent large-scale machine learning study, which detected two anatomical network-based subtypes within the EP population: one starting with volumetric reductions in the hippocampus and related subcortical regions (“early subcortical” subtype) and second, with the initial alterations in insula and Broca's area cortex (“early cortical” subtype; Jiang et al., 2023). These subtypes successfully predicted clinical outcomes, i.e., substantially higher psychosis, depression, and anxiety and general psychopathology scores found in the “early cortical” subtype (Jiang et al., 2023). These findings highlight the capability of MRI approaches to model illness courses; however, prior reports center around volumetric cortical alterations with a limited focus on subcortical regions, including those found in the brainstem.

The midbrain is the uppermost region of the brainstem, which lies at the bottom of the brain, serving as a connector between the spinal cord and the brain. The midbrain and hippocampus show increased resting cerebral blood flow in ultra-high-risk psychosis individuals (Allen et al., 2016). Individuals with schizophrenia also show reduced functional connectivity in the hippocampus→midbrain→striatum network (Gangadin et al., 2021). In addition, PET studies in individuals with schizophrenia demonstrate elevated dopamine synthesis capacity in the substantia nigra, another hub region of the midbrain, which in turn is associated with symptom severity in schizophrenia (Howes et al., 2013). Increased activation of the midbrain in response to neutral stimuli has been suggested as a functional MRI biomarker of delusions in schizophrenia (Romaniuk et al., 2010). The midbrain structure called the ventral tegmental area (VTA) is a critical brain region for reward learning and memory and is impaired in the early stages of psychosis. The VTA→hippocampal functional connectivity is increased in EP, while VTA→striatal connectivity is reduced (Gregory et al., 2021; McHugo et al., 2021), suggesting differential effects of midbrain circuits mediating seemingly contradictory effects in behavior (i.e., hypodopaminergic anhedonia vs hyperdopaminergic delusion or paranoia). Putatively, the midbrain modulates traditional cortical alterations in psychotic disorders in a way that transcends the conventional dopaminergic focus of the midbrain (the midbrain is the brain's center for the synthesis of dopamine). The prior work shows that the midbrain is a critical region affected by EP (Bielawski and Bondurant, 2015) and potentially a crucial player in the functional outcomes of EP and psychotic disorders. Still, only a few studies aim to characterize midbrain's morphological and microstructural alterations in EP.

In this study, we used publicly available data from the HCP-EP, aimed to generate a high-quality, comprehensive dataset in individuals with early-course psychosis (i.e., within 5 years of psychosis onset) to characterize seed-based and voxel-based level alterations in midbrain and their behavior relationships to determine the relevance of midbrain to common alterations in EP and psychotic disorders.

## Materials and Methods

### Participants

The HCP-EP dataset comprises 251 subjects (183 EP and 68 matched healthy controls, Release 1.1). We included subjects with T1w and DTI data (123 EP and 56 controls). The EP cohort consisted of subjects within 5 years of psychosis onset, including affective and nonaffective psychoses. Inclusionary diagnoses were DSM-5 (American Psychiatric Association, 2022) diagnosis of schizophreniform disorder, schizophrenia, schizoaffective disorder, psychosis unspecified type, delusional disorder, or brief psychotic disorder, major depressive disorder with psychotic features (single and recurrent episodes), or bipolar disorder with psychotic features (including most recent episode depressed and manic types), all with onset within 5 years before study entry. We obtained approval from NIH/NIMH through a Data Use Certification agreement (OMB Control Number: 0925-0667).

### MRI

This study focused on the (1) T1w MPRAGE (0.8 mm isotropic resolution) and the (2) diffusion-weighted MRI sequence [multiband acceleration factor of 4, 92 directions in each shell (*b* = 1,500 and 3,000) acquired twice: once with AP and once with PA phase encoding]. More details on the data acquisition parameters can be found on the HCP-EP website. (https://www.humanconnectome.org/storage/app/media/documentation/HCP-EP1.1/Appendix_1_HCP-EP_Release_Imaging_Protocols.pdf)

### Voxel-based morphometry and deformation-based morphometry

We employed Anatomical Normalization Tools (ANTs) for the whole-brain T1 structural analysis. We used 123 EP subjects and 53 controls with complete T1w images that passed quality control procedures. To examine whether results are reproducible over distinct template spaces, the voxel-based morphometry (VBM; Ashburner and Friston, 2000) procedure was executed thrice using three distinct structural atlases (two general atlases and one local template): (1) the common standard template MNI152, (2) OASIS (Marcus et al., 2010), and (3) a local template constructed from 56 healthy controls (ANTs’ antsMultivariateTemplateConstruction2). Each T1w image was registered (ANTs’ antsRegistrionSyN) onto each template, for VBM (Antonopoulos et al., 2023). In addition, we employed deformation-based morphometry (DBM) to capture the spatial variability of shape changes among the deformation fields from image registration (Chung et al., 2001). DBM measures variabilities of the registration movements using the mathematical quantity Jacobian determinant. In contrast, VBM measures the variabilities in image intensity differences and is considered to somewhat overlook spatial shape changes during linear and nonlinear image registration steps. DBM performs a similar statistical analysis to VBM but considers the values of Jacobian determinant, whose value ranges ∼1, indicating regional shrinkage for values <1 and regional expansion for values >1.

### DTI and tract-based spatial statistics

Diffusion data was motion corrected (FSL's eddy), skull stripped (FSL's brain extraction tool), and corrected for field inhomogeneities (FSL's topup). The scan motion was estimated by FSL's eddy and display low motion artifacts (Table 1). We compared results from the data with and without outliers’ correction and found similar outcomes; hence in order to minimize the steps of manipulation, we continue our analysis with the preprocessing steps outlined above. We generated DTI indices (FSL's dtifit): fractional anisotropy (FA), mean diffusivity (MD), and axial diffusivity (AD). Then, the data was registered to the MNI atlas and analyzed for group differences at the voxel level by *t* tests, similar to VBM. Tract-based spatial statistics (TBSS) was performed based on FAs following the FSL's TBSS guidelines (FSL's randomize, 123 EP subjects and 56 controls with complete and adequate DW images; Smith et al., 2006).

**Table 1.** Scan in motion parameters

	OL%/sub	# sub OL >3%	GP cor OL%/sub	# sub GP cor OL >3%
EP	0.7%	2	0.3%	1
Controls	0.3%	0	0.2%	0

The average percentage of outliers per subject (OL%/sub) is low for EP subjects and controls, and the total number of subjects with outliers larger than 3% were only 2 in the EP group. Applying a Gaussian process correction to all outliers slides reduced this numbers marginally without a significant change in results. OL, outliers; sub, subject; GP, Gaussian process; cor, corrected.

### Midbrain-focused analyses

Given the vital role of the midbrain in psychotic disorders pathophysiology, we segmented the midbrain region of interest (ROI) using FreeSurfer and manually removed the portions corresponding to the pons and medulla. We used the first slice where there was a clear distinction of the pons as a marker of the inferior portion of the midbrain. This segmented midbrain structure was then registered to all DTI scalar maps for seed-based analysis of DTI indices.

### Symptom data

The Positive and Negative Syndrome Scale (PANSS; Kay et al., 1987) and the Clinical Assessment Interview for Negative Symptoms (CAINS; Kring et al., 2013) were obtained as part of the behavioral dataset of the HCP-EP. Both scales are well validated, reliable, and extensively utilized in the assessment of current symptom severity in psychosis populations, including EP. Each section of the PANSS test displays an internal reliability of 0.73, 0.83, and 0.79 for positive, negative, and general psychopathology scales, respectively, and interrater reliability of *r* = 0.83, 0.85, 0.87 for positive, negative, and general psychopathology scales, respectively (Kay et al., 1988); meanwhile, the CAINS has an internal reliability of 0.76 and test–retest reliability of *r* = 0.69. (Kring et al., 2013)

### Statistical analyses

VBM and DBM statistics were completed in Python using Numpy and Scipy packages with voxel-wise *t* tests (alpha = 0.05) followed by familywise error rate correction using the false discovery rate. FA, AD, and MD map voxel-level *t* tests (alpha = 0.05) were completed using the same Python-based approach. Seed-based midbrain outcomes were analyzed using Welch two-sample *t* tests on the mean FA, AD, and MD maps in R with the *t* test function and shown with a violin plot using the ggplot function. The TBSS was carried out on FA maps, in which the general linear model steps were done using the FSL randomise function with 1,000 permutations. The correlation analyses examined associations between the symptom scales (PANSS and CAINS), volume measures (i.e., whole-brain Jacobian determinant, midbrain volumes), and DTI indices in the EP group in R using paris.panels from the package PerformanceAnalytics.

## Results

The total dataset consisted of 179 subjects (EP and controls), with 68.7% being males and 31.3% females, evenly distributed in EP (males, 61%; females, 37.4%) and controls (males, 64.3%; females, 35.7%) subsets (Table 2). Subjects were primarily right-handed (EP, 85.4%; controls, 80.4%). The EP cohort included 75.6 and 24.4% nonaffective and affective psychosis subjects, respectively.

**Table 2.** Demographic characteristics of the HCP-EP dataset

	EP, *n* = 123	Controls, *n* = 56
Age, years, mean (SD)	23.06 (3.64)	24.43 (4.27)
Sex, *n* (%)	M: 75 (61.0%)	M: 36 (64.3%)
	F: 46 (37.4%)	F: 20 (35.7%)
	N/A: 2 (1.6%)	
Handedness, *n* (%)	Right: 105 (85.4%)	Right: 45 (80.4%)
	Left: 10 (8.1%)	L: 10 (17.8%)
	Ambidextrous: 5 (4.1%)	Ambidextrous: 1
	N/A: 3 (2.4%)	(1.8%)
Psychosis phenotype, *n* (%)	Affective: 28 (24.4%)	NA
	Nonaffective: 93 (75.6%)	
Lifetime antipsychotic medication exposure: months, mean (SD)	Yes: 97 (78.9%)	NA
	No: 26 (21.1%)	
	14.06 (15.58)	
Race/ethnicity, *n* (%)	Asian: 9 (7.3%)	Asian: 7 (12.5%)
	Black: 45 (36.5%)	Black: 5 (8.9%)
	Hispanic/Latino: 4 (3.2%)	Multiracial: 9 (16.1%)
	Multiracial: 7 (5.6%)	White: 34 (60.7%)
	White: 58 (47.1%)	N/A: 1 (1.8%)
	N/A: 2 (1.6%)	

One EP subject did not have demographic data reported.

**Figure 1.**
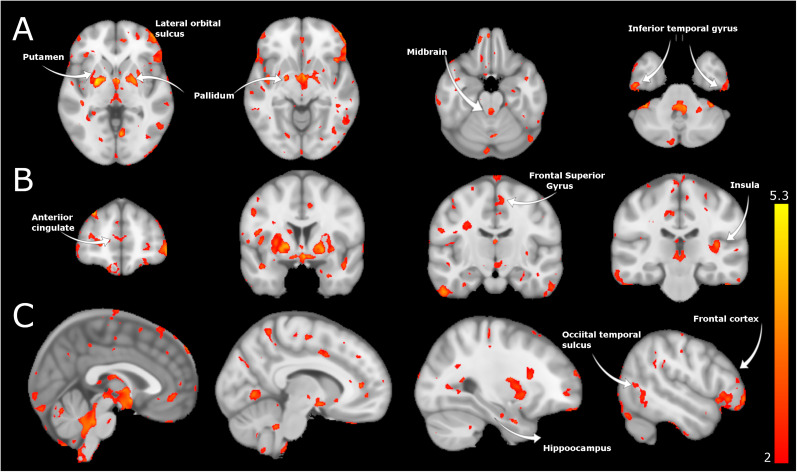
Volumetric differences in the EP versus healthy control groups. Volumetric differences are estimated from the DBM parameter using the Jacobian determinant results. Volumetric differences are shown with (***A***) axial sections, (***B***) coronal sections, and (***C***) sagittal sections. Yellow represents voxel-wise volume increases, and red represents reductions in EP versus healthy controls (*t* > 2.2, *q* < 0.05).

The DBM method is agnostic to tissue type and relies on image contrast for displacements in the registration; therefore, DBM does not suffer from tissue misclassification as VBM and is potentially more sensitive than VBM to subtle alterations comparing healthy and patient populations (Manera et al., 2019). DBM revealed significant between-group morphological alterations, indicating higher displacement (i.e., Jacobian determinant values) in frontal regions and the superior regions within the midbrain in EP versus healthy controls (Fig. 1). In contrast, the standard VBM yielded no significant between-group differences. All three templates produced similar patterns, indicating the reproducibility of volumetric alterations in EP versus controls and highlighting the same regional alterations throughout the brain independent of the segmentation (i.e., tissue classification) template. In addition, we could show DBM alterations in the hippocampus, frontal cortex, insula, putamen, and cerebellum (Fig. 1).

**Figure 2.**
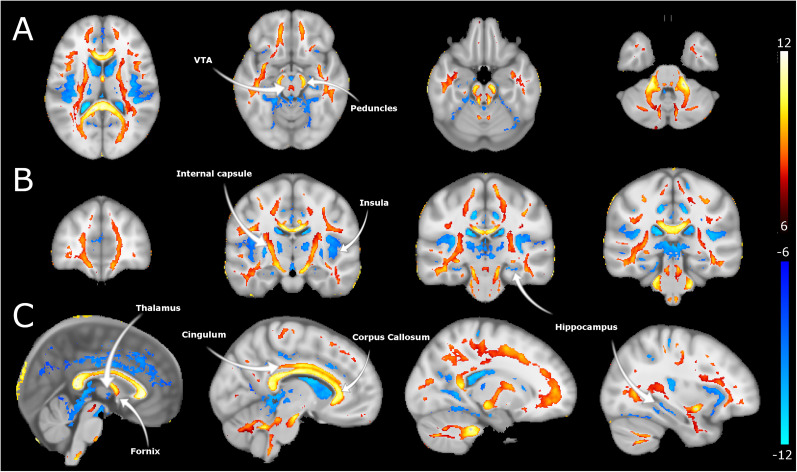
Fractional anisotropy differences in the EP versus healthy control groups. ***A***, Axial, (***B***) coronal, and (***C***) sagittal sections of FA alterations between EP and controls. Differences are estimated from the FA maps registered to MNI space and following a *t* test. Hot colors represent reductions in FA, while cool colors refer to increases in FA between healthy controls and EP cohorts larger than *t* > 6 and *q* < 0.0001.

**Figure 3.**
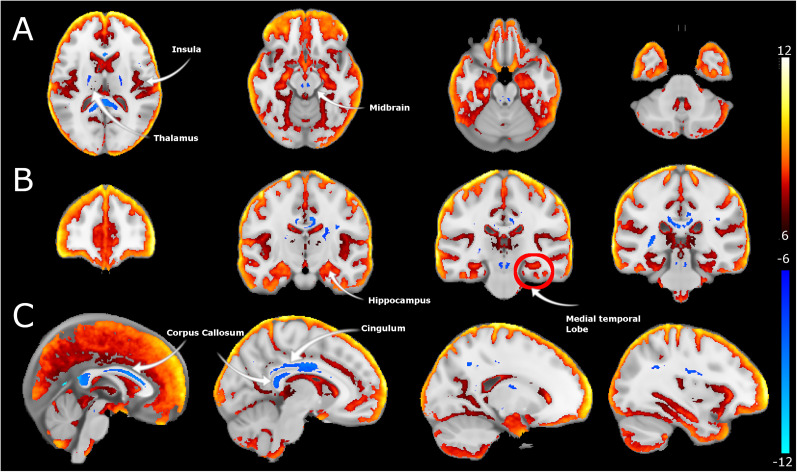
Mean diffusivity alterations in EP. ***A***, Axial, (***B***) coronal, and (***C***) sagittal sections of FA alterations between EP and controls. Differences in MD are estimated from the MD maps registered to MNI space and following a *t* test. Hot colors represent reductions in MD, while cool colors refer to increases between healthy controls and EP cohorts larger than *t* > 6 and *q* < 0.0001.

**Figure 4.**
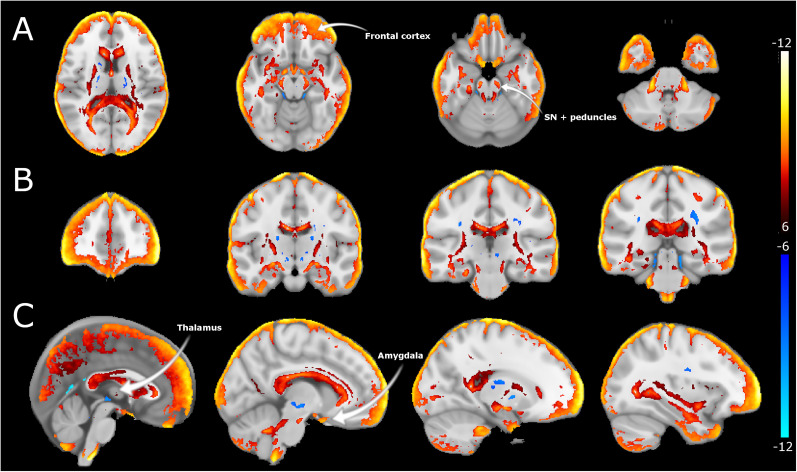
Axial diffusivity differences in EP. ***A***, Axial, (***B***) coronal, and (***C***) sagittal sections of AD alterations between EP and controls. Differences in AD are estimated from the AD maps registered to MNI space and following a *t* test. Hot colors represent reductions in AD, while cool colors refer to increases between healthy controls and EP cohorts larger than *t* > 6 and *q* < 0.0001.

To determine alterations in white matter microstructure between EP and controls, we obtained DTI indices: whole-brain maps of FA, AD, and MD. Susceptibility artifacts were modeled by AP and PA acquisition and corrected to improve image quality. FA maps registered to the MNI152 template revealed significant between-group differences in several white matter tracts (corpus callosum, cingulum, and internal capsule), with lower FA in EP versus controls (Fig. 2). The voxel-based analysis shows alterations in voxels in the midbrain; significantly lower FA indices were seen at the cerebral peduncles and regions adjacent to VTA in EP versus controls, as shown in Figure 2. MD maps showed substantially higher (e.g., in the corpus callosum, midbrain, and basal ganglia regions) and lower (in the medial temporal lobe) MD in EP versus controls. The MD alterations in the midbrain were localized to the VTA area (Fig. 3). The AD maps displayed between-group differences mainly in the ventricular and peripheral CSF spaces, with lower AD in EP versus controls (Fig. 4). Notably, the AD results also indicated alterations in the midbrain regions (i.e., in peduncles and areas adjacent to VTA and substantia nigra), like those captured in FA maps. All DTI indices display significant alterations in the cerebellum.

**Figure 5.**
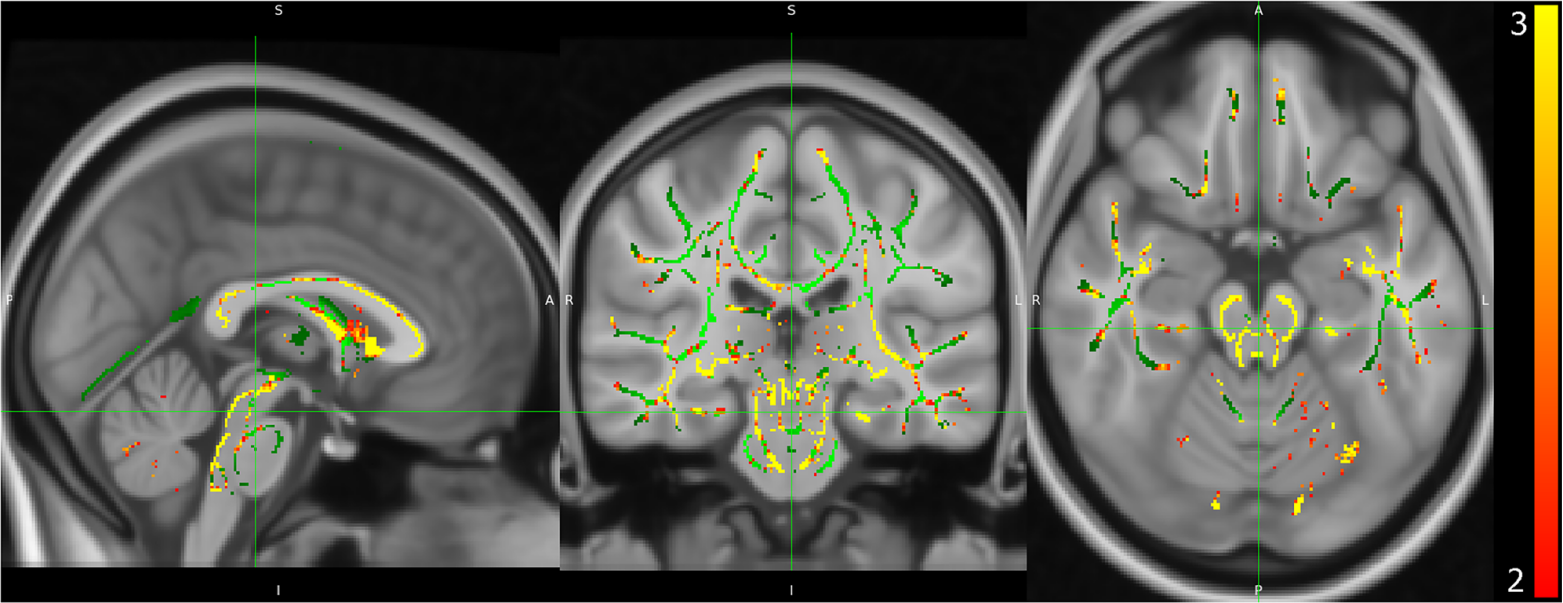
White matter alterations in EP. TBSS maps report differences from the corrected *t* test following TBSS pipelines. Heat maps represent relative differences between healthy controls and EP cohorts larger than *t* > 2 and *q* < 0.05.

In addition to the DTI indices, we carried out TBSS to extract more granular information about major white matter tracts. TBSS projects tract data onto an artificially constructed “skeleton” (Fig. 5, green) of all white matter tracts. TBSS then performs a statistical analysis of the FA maps over this “skeleton.” TBSS results revealed significantly higher FA values in EP than controls in multiple tracks, with the highest effect sizes in the midbrain, cingulum, and corpus callosum. TBSS highlighted significant FA reductions in white matter tracts within the midbrain in EP versus controls.

**Figure 6.**
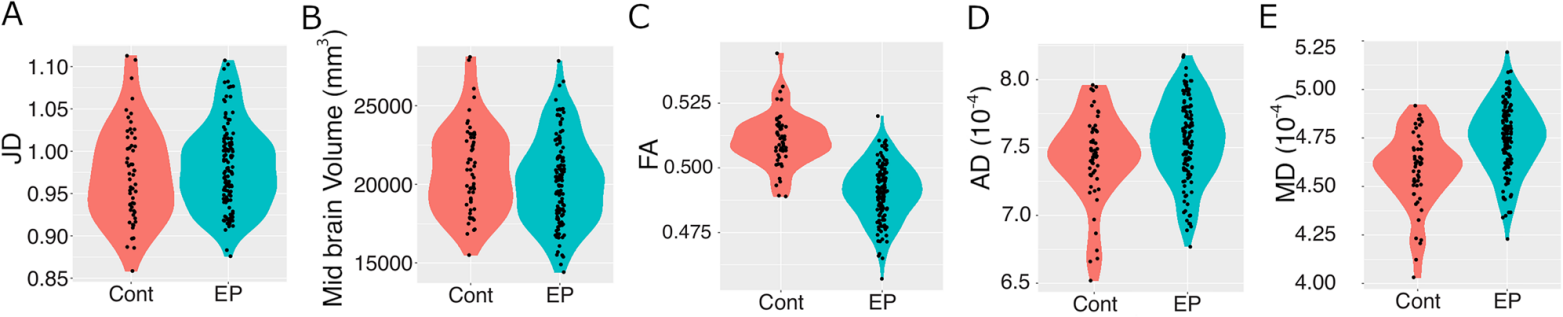
The grouped subfigures are violin plots of the distributions of midbrain microstructural alterations in EP versus healthy controls in ROI means of Jacobian determinant (***A***), midbrain ROI volume (***B***), and ROI means of FA (***C***), AD (***D***), and MD (***E***) maps. And their Welch two-group *t* test results, respectively.

As the next step, a midbrain ROI was generated from FreeSurfer v.6.0. Destrieux atlas (https://mindboggle.info/data.html; Klein and Tourville, 2012). A seed-based analysis used the midbrain ROI on the Jacobian determinant, midbrain volumes, FA, MD, and AD to determine specific midbrain macro- and microstructural differences between EP and controls. The results showed no significant differences in the measures of Jacobian determinant (*t* = 0.96, *p* = 0.33) or native midbrain volumes (*t* = −1.61, *p* = 0.11). However, there were significant between-group differences in FA (*t* = −11.58, *p* = 2.2 × 10^−16^), AD (*t* = 2.33, *p* = 0.02), and MD (*t* = 5.23, *p* = 1.06 × 10^−6^) in the midbrain (Fig. 6).

**Figure 7.**
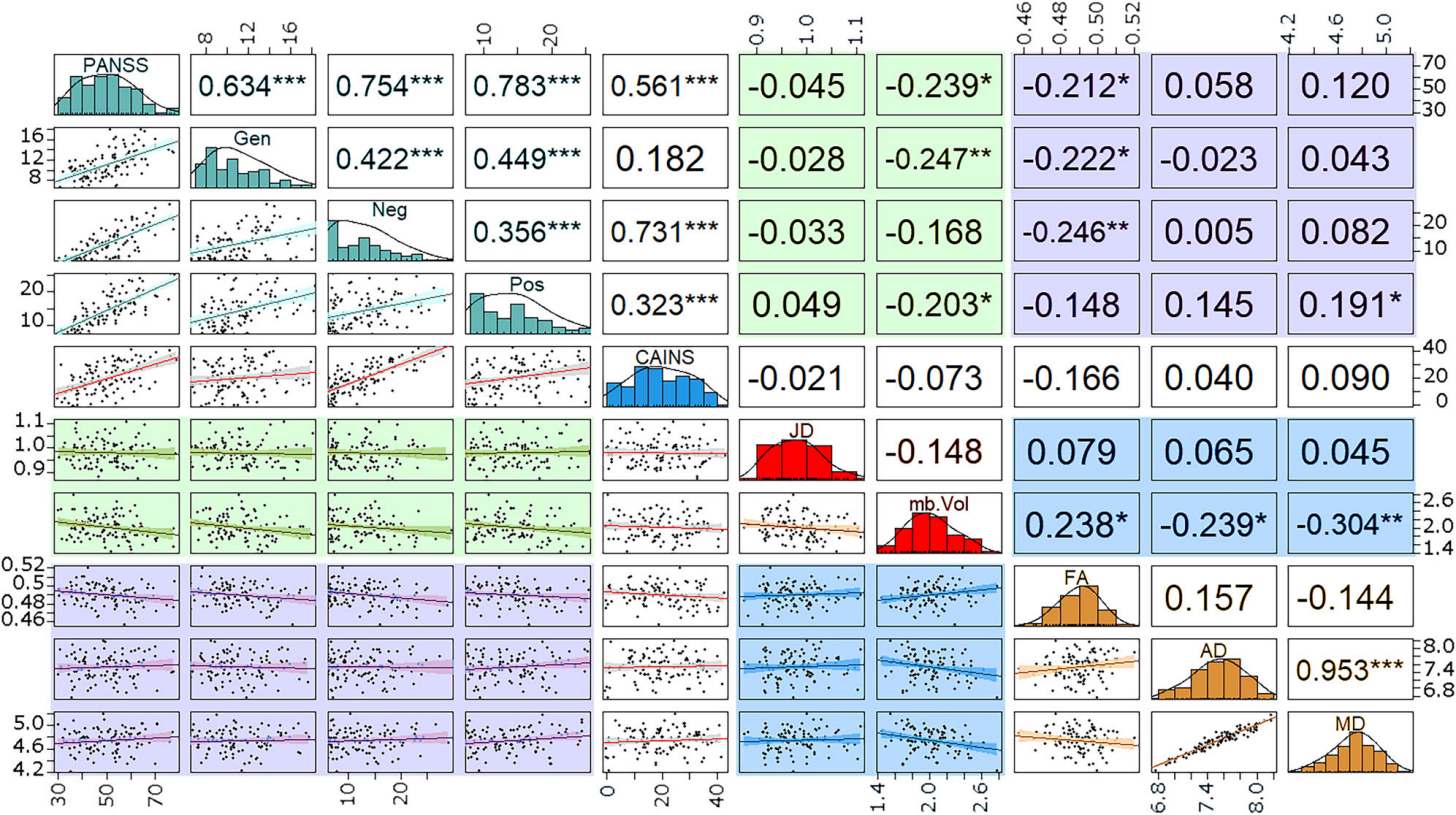
Relationships between the PANSS and CAINS symptom scores and Jacobian determinant, midbrain volumes, and DTI indices of midbrain microstructure. The diagonal shows the distribution of each score (PANSS, CAINS, volumetrics, or DTI). Highlighted portions: turquoise, PANSS scores; red, T1 W volumetrics; orange, DTI measures; green, volumetrics versus PANSS; purple, DTI versus PANSS; blue, volumetrics versus DTI. The bottom quadrant shows the scatterplots with fitted correlation, and the top quadrant shows *r*^2^ of the correlation (*p < 0.05, **p < 0.01, and ***p < 0.005). The midbrain volume scale is given by #×10^4^ and the AD and MD are in the #×10^−4^ for ease of reading.

The symptom metrics (i.e., PANSS and CAINS) displayed significant correlations between themselves, except for CAINS and the PANSS General Psychopathology subscale (*r*^2^ = 0.18, *p* = 0.056, trend). Among the MRI outcomes (i.e., ROI means of Jacobian determinant, midbrain volumes, and the mean of DTI indices), the midbrain volume negatively correlated with MD (*r*^2^ = −0.30, *p* = 0.001), AD (*r*^2^ = −0.24, *p* = 0.012), and FA (*r*^2^ = −0.238, *p* = 0.012). The midbrain AD correlated with MD (*r*^2^ = 0.953, *p* < 0.001), but FA did not correlate with either (Fig. 7). The Jacobian determinant did not correlate with any other MRI metrics.

The correlation analyses between symptom scores (i.e., PANSS, CAINS) and MRI-derived measures (i.e., volumetrics and DTI) showed significant correlations between midbrain volume and symptom severity. The midbrain volume displayed significant negative correlations with the PANSS total score (*r*^2^ = −0.239, *p* = 0.011), Positive Symptoms subscale score (*r*^2^ = −0.203, *p* = 0.032), General Psychopathology subscale score (*r*^2^ = −0.247, *p* = 0.009), and the trend-level negative correlation with the Negative symptoms’ subscale score (*r*^2^ = −0.168, *p* = 0.077). The CAINS did not correlate with any of the MRI-derived measures. The midbrain FA showed significant negative correlations with the PANSS total score (*r*^2^ = −0.212, *p* = 0.026), General Psychopathology score (*r*^2^ = −0.222, *p* = 0.019), and Negative symptoms score (*r*^2^ =  0.246, *p* = 0.009); in addition, there was a trend for the CAINS score (*r*^2^ = −0.166, *p* = 0.081). The MD showed a significant positive correlation with the PANSS Positive symptoms score (*r*^2^ = 0.191, *p* = 0.045). The AD did not correlate with any symptom metric.

## Discussion

This study consistently identified midbrain alterations using the whole-brain and region-specific analyses of volumetric, microstructure, and connectivity analysis in the publicly available HCP-EP sample. Alterations in midbrain were accompanied by widespread cortical and white matter alterations in frontal, temporal, and cingulate cortical regions, cerebellum, corticospinal, and corpus callosum white matter tracts. DBM shows significant differences around the midbrain and hippocampus. The DTI analysis displays extensive FA, AD, and MD alterations in several brain regions and white matter tracts, especially around the midbrain, confirmed by a seed-based analysis of DTI metrics. The midbrain volume in EP subjects significantly correlated with several behavior indices and all DTI indices. The correlation analyses determined that FA midbrain values are associated with the severity of positive, negative, and general psychopathology PANSS-based symptoms. TBS, as a proxy of altered connectivity, revealed deep WM and midbrain tract alterations in EP subjects, mainly in the corpus callosum, cingulum, and cerebral peduncles white matter tracts.

Several reports describe brain structure volumetric alterations in EP subjects. In addition to DBM changes to midbrain, we highlight medial temporal lobe alterations (i.e., the hippocampus), striatum, and insula consistent with prior DBM findings in first-episode schizophrenia individuals (Torres et al., 2016). EP reductions have been reported in the frontotemporal region and left postcentral gyrus (Kolenič et al., 2020), hippocampus (Briend et al., 2020; McHugo et al., 2021, 2024; Brunner et al., 2022), the temporal cortex, frontal cortex (Yang et al., 2022), precentral cortex, and insula (Garcia-Marti et al., 2023). EP subjects also display volume reductions in precuneus, uncus, and amygdala (Canal-Rivero et al., 2020). These alterations have been confirmed not only in volume but also in cortical thickness. In a first-episode psychosis cohort, it was found that cortical thickness, surface area, and subcortical volumes display significant variability, and the researchers propose further examination to determine trajectories (Antoniades et al., 2021). EP subjects show clusters of reduced white matter and lower GM volume in the left median cingulate cortex, cerebellum, inferior parietal gyrus, and thalamus (Si et al., 2024). The thalamus displays volume reductions coinciding with microstructural (assessed with DTI) alteration in EP subjects (Alemán-Gómez et al., 2020, 2023). Our work largely confirms prior reports and positions the midbrain as an altered foci early in the psychotic disorders and EP.

Alterations in gross anatomy also imply microscopic alterations in EP. A cohort of clinical high-risk individuals shows cerebellum differences in FA and radial diffusivity (Fitzsimmons et al., 2020). Reductions in FA along white matter tracts connecting the limbic striatum with the limbic cortical network and the anterior external capsule segment connections to the right prefrontal cortex are a proxy for psychosis risk (Straub et al., 2020). Ultra-high-risk psychosis subjects show increased radial diffusivity in the left anterior thalamic radiation and reduced bilateral thickness across the frontal, temporal, and parietal cortices (Tomyshev et al., 2019). EP individuals exhibit lower FA in the fornix, smaller hippocampal volumes (Baumann et al., 2016), and reduced mean kurtosis values in the thalamic regions (Cho et al., 2019). There is a significant microstructural asymmetry of the superior longitudinal fasciculus in clinical high risk for developing psychosis females compared with control females. Still, there is no hemispheric difference between clinical high-risk for developing psychosis versus control males (Steinmann et al., 2021). The structural alterations in EP are even present from an early age. Young subjects with subclinical psychotic experiences show lower mean and radial diffusivity in the arcuate fasciculus (Dooley et al., 2020). EP subjects also display alterations in quantitative anisotropy in the corpus callosum, cerebellum, and peduncles (Moghaddam et al., 2024). We validated these discrete findings in a model-free approach using a voxel-based analysis of DTI indices. Lower FA in regions proximal to the superior longitudinal fasciculus and corticospinal tract bilaterally and in the left inferior frontal-occipital fasciculus and inferior longitudinal fasciculus are related to higher psychotic episodes at baseline (DeRosse et al., 2017). The myriads of microstructural alterations over disparate brain regions observed in our study and discussed here indicate that network-level mechanism may be a potential biomarker for EP that may provide relevant insights about functional alterations in EP and the midbrain also is a critical player in this dysfunction.

The structural alterations in EP are associated with behavioral alterations in EP as we describe in this study. Prior EP studies suggest that alterations in regional brain activity, gray and white matter structure, and connectivity precede and predict the psychosis onset (Keshavan et al., 2011; Schobel et al., 2013; Cao et al., 2019; Lewandowski et al., 2020). These early brain-based disease markers are associated with cognitive and symptom manifestations in clinical high-risk/EP populations (Jalbrzikowski et al., 2021). Frontal and parietal cortical volume loss EP is associated with working memory disruptions (Rapado-Castro et al., 2021). The mean kurtosis in the thalamus and the orbitofrontal cortex correlated with spatial working memory accuracy in EP subjects; in contrast, no such significant correlations were observed in healthy subjects (Cho et al., 2019). The laterality index of superior longitudinal fasciculus III for clinical high-risk developing psychosis females correlated negatively with poorer working memory functioning (Steinmann et al., 2021). Prior studies point to reduced FA and higher MD in the superior longitudinal fasciculus and inferior frontal-occipital fasciculus in EP, and these are associated with hallucination severity (Sato et al., 2021). Processing speed in EP is associated with the structural connectivity of superior frontal gyri, precuneus, somatomotor, temporal-mesial cortices, thalamus, superior-parietal cortex, caudate, pallidum, and lateral-occipital cortex, and clinical subtypes of EP are characterized by distinct brain-connectivity profiles (Griffa et al., 2019). In our study, we also show that midbrain is associated clinical markers of PANSS implying that midbrain alterations are also relevant to the development of the disease. The reported associations between brain structure and symptoms suggest that the EP alterations in brain changes are putative markers of EP development and the midbrain could be an equally relevant structure in the etiology of EP.

The significance of the midbrain could be related to a dopamine-related alteration. With the HCP-EP data cannot determine the validity of this assertion but is a reasonable candidate since dopamine is an extensively studied target in psychotic disorders since most approved treatments focus on blocking D2 action. The midbrain is the primary source of dopamine synthesis, suggesting it could be an essential rain region that alters and mediates network changes in EP. The dopamine synthesis and signaling alterations in the midbrain and striatum are downstream effects from the primary glutamatergic and GABAergic from the upstream limbic system (Lisman et al., 2008; Lodge and Grace, 2011), suggesting a multiple systems disorder and close relationship between the hippocampus and midbrain that may extend beyond dopamine. In fact, midbrain dopamine neurons inhibit striatum by the release of GABA which may also contribute to our observed changes due to membrane uptake of GABA (Tritsch et al., 2014). Additionally, norepinephrine which is synthesized in the pons from dopamine can also inhibit dopamine neurons in midbrain (Paladini and Williams, 2004). Even though multiple candidates are mechanistic targets for the observed alteration of midbrain in EP, another avenue may be in network brain changes.

Our results consistently observed several alterations in the midbrain and hippocampus. In our multimodal analyses, morphological, microstructural, and tract-based alterations in the midbrain emerged as a critical hub in EP. However, others could be equally important in the etiology of psychotic disorder. A network modeling provides insights about two putative trajectories of psychotic disorders, highlighting two distinct regional starting points. One trajectory starts in Broca's area and the insula, then propagates into frontal and medial temporal regions, and concludes in the cerebellum and subcortical areas (Jiang et al., 2023). The second trajectory initiates in the hippocampus and amygdala; then the parahippocampus, thalamus, and nucleus accumbens; then the caudate and insula; then the putamen; and concludes in several cortical areas (Jiang et al., 2023). Most of these areas were observed in our study, suggesting that midbrain may be an important target, and it must be included to determine distinct phenotypes of psychotic disorders. The network modeling also shows that the structural alteration correlates with PANSS positive and negative symptoms (Moghaddam et al., 2024) as we report for midbrain structural features. The network modeling of Jiang et al. (2023) also relied on VBM analysis, which usually suffers from potential limits from its segmentation step. Even though we make the argument of DA and midbrain due to its understudied nature and putative relevance because of currently approved treatments, we have to recognize other regional markers that have been implicated in schizophrenia, such as serotonin hyperactivity(Mahdiar et al., 2023) and alterations in neuroinflammatory signaling via increases in IL6 and TNFα (Purves-Tyson et al., 2019) that may be important players in the distributed alterations among several brain regions reported in this article.

Several limitations of the study should be noted. First, the cross-sectional nature of the HCP-EP sample limits our ability to examine the structural brain alterations along the specific epochs/trajectories of evolving psychosis, e.g., “early” clinical high risk versus “late” clinical high risk versus EP. One of the strengths of our analyses is its multimodal approach, which incorporates DBM, seed-based volume analysis, voxel-based DTI analysis, and seed-based analysis of DTI metrics. However, other diffusion approaches, such as NODDI (Zhang et al., 2012), may provide more specific markers of the microstructural basis of the alterations in DTI metrics. This study focused on brain structure alterations in EP; hence, the potential alterations in functional networks have not been examined. Our future work will expand these findings to (1) determine the topological alterations in brain structure using tractography and volume analysis of white matter tracts associated with psychotic disorders, (2) sex differences in EP, and (3) functional connectivity analysis of resting state networks.

In conclusion, our results suggest that the midbrain structure, microstructure, and connectivity is altered in EP and we posit that it may be a strong contributor to a future putative “biomarker profile” (Mirzakhanian et al., 2014) of EP. Ongoing work highlights the altered brain network hypothesis but usually omits the midbrain. Our study presents evidence of macro- and microstructural midbrain alterations in the early-course phase of psychotic disorders co-occurring with several brain regions that have gained more attention (e.g., hippocampus, insula, and frontal cortex). Employing an assay of multimodal MRI with the high-quality, large, publicly available dataset (HCP-EP), EP subjects show that alterations in frontal and temporal (Keshavan and Amirsadri, 2007) regions were concomitant with midbrain highlighting its prominence in EP, leading us to advocate for its role in the network-level alteration early in the development of psychotic disorders.

## Data availability

Data can be obtained through an NDA with NIMH. Instruction and relevant information can be found https://www.humanconnectome.org/study/human-connectome-project-for-early-psychosis/document/hcp-ep

## Declarations

Data used in the preparation of this manuscript was obtained from the National Institute of Mental Health Data Archive. The National Institute of Mental Health Data Archive is a collaborative informatics system created by the National Institutes of Health to provide a national resource to support and accelerate research in mental health. Dataset identifier(s): National Institute of Mental Health Data Archive Collection #2914. This manuscript reflects the views of the authors and may not reflect the opinions or views of the NIH or of the Submitters submitting original data to National Institute of Mental Health Data Archive DBM.

## Synthesis

Reviewing Editor: Jibran Khokhar, Western University Department of Anatomy and Cell Biology Decisions are customarily a result of the Reviewing Editor and the peer reviewers coming together and discussing their recommendations until a consensus is reached. When revisions are invited, a fact-based synthesis statement explaining their decision and outlining what is needed to prepare a revision will be listed below. The following reviewer(s) agreed to reveal their identity: Elvisha Dhamala. Note: If this manuscript was transferred from JNeurosci and a decision was made to accept the manuscript without peer review, a brief statement to this effect will instead be what is listed below.

Zhou and colleagues investigate structural brain alterations in early psychosis (EP) and their associations with clinical outcomes, using a comprehensive set of imaging techniques, including VBM, DBM, DTI, and TBSS. The study explores grey and white matter differences between EP and control groups, aiming to uncover potential biomarkers for psychosis. While the study provides valuable descriptive insights and includes a large sample size, several methodological concerns, particularly related to the statistical robustness of the findings, weaken the overall conclusions. Furthermore, the manuscript emphasizes the midbrain in the discussion, though structural differences are observed across the brain.

Major Concerns:

1. Lack of Sensitivity/Specificity Analysis:

- The study lacks an assessment of the sensitivity and specificity of brain measures differentiating EP and neurotypical groups, despite being framed as a search for biomarkers.

2. Unclear Link to Mechanistic Framework:

- The discussion speculates on a link between midbrain findings and dopaminergic alterations, but does not provide a clear mechanistic argument for dopamine being the most relevant neurotransmitter over others, such as serotonin or noradrenaline. The functional anatomy of midbrain findings is not sufficiently explored.

3. Statistical Inference Concerns:

- There is insufficient correction for multiple comparisons, particularly in the correlations between brain structure and symptom scales. Furthermore, the rationale for using a false-discovery rate (FDR) approach with a low t-threshold is unclear, raising concerns about the presence of false positives.

4. Inconsistent Thresholding in Figures:

- There are discrepancies in the t-thresholds used across figures, and it is unclear why a permutation approach was not consistently applied across different metrics. Additionally, the study lacks a formal assessment of effect consistency across imaging modalities.

5. Unaddressed Motion Artifacts:

- The manuscript does not provide details on how in-scan motion, which could potentially confound group differences, was accounted for.

Minor Concerns:

1. Confusing Results Presentation:

- Figures 1-4 lack calibrated color scales, and certain figures are difficult to interpret due to missing labels (e.g., Figure 6 lacks y-axis labels).

2. Contradictory Claims About MRI Biomarkers:

- The introduction states that MRI biomarkers may only apply to a small subset of individuals (6%), yet argues that developing these biomarkers is a high priority. These statements need clarification to avoid contradiction.

3. Acronym Overuse:

- The manuscript employs numerous acronyms, which can impede readability. A reduction in unnecessary acronyms is recommended to enhance clarity.

4. Noise in Results:

- The results appear noisy, with significant differences in small, scattered regions. It is unclear whether thresholding was used to minimize noise, and if not, the authors should justify this.

5. Figure 7 Readability:

- Figure 7, along with supplementary figures, has inconsistent font sizes for r-squared values and lacks clear axis labels, making it difficult to interpret. Improvements in figure clarity are needed.

## References

[B1] Alemán-Gómez Y, et al. (2020) Partial-volume modeling reveals reduced gray matter in specific thalamic nuclei early in the time course of psychosis and chronic schizophrenia. Hum Brain Mapp 41:4041–4061. 10.1002/hbm.25108 33448519 PMC7469814

[B2] Alemán-Gómez Y, et al. (2023) Multimodal magnetic resonance imaging depicts widespread and subregion specific anomalies in the thalamus of early-psychosis and chronic schizophrenia patients. Schizophr Bull 49:196–207. 10.1093/schbul/sbac113 36065156 PMC9810016

[B3] Allen P, Chaddock CA, Egerton A, Howes OD, Bonoldi I, Zelaya F, Bhattacharyya S, Murray R, McGuire P (2016) Resting hyperperfusion of the hippocampus, midbrain, and basal ganglia in people at high risk for psychosis. Am J Psychiatry 173:392–399. 10.1176/appi.ajp.2015.15040485 26684922

[B4] Antoniades M, Haas SS, Modabbernia A, Bykowsky O, Frangou S, Borgwardt S, Schmidt A (2021) Personalized estimates of brain structural variability in individuals with early psychosis. Schizophr Bull 47:1029–1038. 10.1093/schbul/sbab005 33547470 PMC8266574

[B5] Antonopoulos G, More S, Raimondo F, Eickhoff SB, Hoffstaedter F, Patil KR (2023) A systematic comparison of VBM pipelines and their application to age prediction. Neuroimage 279:1–14. 10.1016/j.neuroimage.2023.120292 37572766 PMC10529438

[B6] Ashburner J, Friston KJ (2000) Voxel-based morphometry–the methods. Neuroimage 11:805–821. 10.1006/nimg.2000.0582 10860804

[B7] Baumann PS, et al. (2016) Impaired fornix–hippocampus integrity is linked to peripheral glutathione peroxidase in early psychosis. Transl Psychiatry 6:e859–e859. 10.1038/tp.2016.117 27459724 PMC5545707

[B8] Bielawski M, Bondurant H (2015) Psychosis following a stroke to the cerebellum and midbrain: a case report. Cerebellum Ataxias 2. 10.1186/s40673-015-0037-8 26664729 PMC4673856

[B9] Blackman G, et al. (2023) Prevalence of neuroradiological abnormalities in first-episode psychosis: a systematic review and meta-analysis. JAMA Psychiatry 80:1047–1054. 10.1001/jamapsychiatry.2023.2225 37436735 PMC10339221

[B10] Blessing EM, Murty VP, Zeng B, Wang J, Davachi L, Goff DC (2020) Anterior hippocampal-cortical functional connectivity distinguishes antipsychotic naïve first-episode psychosis patients from controls and may predict response to second-generation antipsychotic treatment. Schizophr Bull 46:680–689. 10.1093/schbul/sbz076 31433843 PMC7147586

[B11] Braun U, et al. (2016) Dynamic brain network reconfiguration as a potential schizophrenia genetic risk mechanism modulated by NMDA receptor function. Proc Natl Acad Sci U S A 113:12568–12573. 10.1073/pnas.1608819113 27791105 PMC5098640

[B12] Briend F, Nelson EA, Maximo O, Armstrong WP, Kraguljac NV, Lahti AC (2020) Hippocampal glutamate and hippocampus subfield volumes in antipsychotic-naive first episode psychosis subjects and relationships to duration of untreated psychosis. Transl Psychiatry 10. 10.1038/s41398-020-0812-z 32398671 PMC7217844

[B13] Brunner G, Gajwani R, Gross J, Gumley AI, Krishnadas R, Lawrie SM, Schwannauer M, Schultze-Lutter F, Fracasso A, Uhlhaas PJ (2022) Hippocampal structural alterations in early-stage psychosis: specificity and relationship to clinical outcomes. Neuroimage Clin 35:1–8. 10.1016/j.nicl.2022.103087 35780662 PMC9421451

[B14] Canal-Rivero M, Tordesillas-Gutiérrez D, Ruiz-Veguilla M, Ortiz-García de la Foz V, Cuevas-Esteban J, Marco de Lucas E, Vázquez-Bourgon J, Ayesa-Arriola R, Crespo-Facorro B (2020) Brain grey matter abnormalities in first episode non-affective psychosis patients with suicidal behaviours: the role of neurocognitive functioning. Prog Neuropsychopharmacol Biol Psychiatry 102:1–8. 10.1016/j.pnpbp.2020.109948 32305356

[B15] Cannon TD, et al. (2015) Progressive reduction in cortical thickness as psychosis develops: a multisite longitudinal neuroimaging study of youth at elevated clinical risk. Biol Psychiatry 77:147–157. 10.1016/j.biopsych.2014.05.023 25034946 PMC4264996

[B16] Cao H, et al. (2019) Altered brain activation during memory retrieval precedes and predicts conversion to psychosis in individuals at clinical high risk. Schizophr Bull 45:924–933. 10.1093/schbul/sby122 30215784 PMC6581134

[B17] Cho KIK, Kwak YB, Hwang WJ, Lee J, Kim M, Lee TY, Kwon JS (2019) Microstructural changes in higher-order nuclei of the thalamus in patients with first-episode psychosis. Biol Psychiatry 85:70–78. 10.1016/j.biopsych.2018.05.019 29961564

[B18] Chopra S, et al. (2023) Network-based spreading of gray matter changes across different stages of psychosis. JAMA Psychiatry 80:1246–1257. 10.1001/jamapsychiatry.2023.3293 37728918 PMC10512169

[B19] Chung MK, Worsley KJ, Paus T, Cherif C, Collins DL, Giedd JN, Rapoport JL, Evans AC (2001) A unified statistical approach to deformation-based morphometry. Neuroimage 14:595–606. 10.1006/nimg.2001.0862 11506533

[B20] DeRosse P, Ikuta T, Karlsgodt KH, Peters BD, Gopin CB, Szeszko PR, Malhotra AK (2017) White matter abnormalities associated with subsyndromal psychotic-like symptoms predict later social competence in children and adolescents. Schizophr Bull 43:152–159. 10.1093/schbul/sbw062 27190281 PMC5216847

[B21] American Psychiatric Association (2022) Diagnostic and statistical manual of mental disorders. Diagnostic and Statistical Manual of Mental Disorders.

[B22] Dooley N, et al. (2020) Psychotic experiences in childhood are associated with increased structural integrity of the left arcuate fasciculus – a population-based case-control study. Schizophr Res 215:378–384. 10.1016/j.schres.2019.08.022 31495700

[B23] Fitzsimmons J, et al. (2020) Cingulum bundle abnormalities and risk for schizophrenia. Schizophr Res 215:385–391. 10.1016/j.schres.2019.08.017 31477373

[B24] Gangadin SS, Cahn W, Scheewe TW, Hulshoff Pol HE, Bossong MG (2021) Reduced resting state functional connectivity in the hippocampus-midbrain-striatum network of schizophrenia patients. J Psychiatr Res 138:83–88. 10.1016/j.jpsychires.2021.03.041 33836433

[B25] Garcia-Marti G, et al. (2023) Progressive loss of cortical gray matter in first episode psychosis patients with auditory hallucinations. Schizophr Res 267:534–535. 10.1016/j.schres.2023.11.011 38044223

[B26] Gregory DF, Rothrock JM, Jalbrzikowski M, Foran W, Montez DF, Luna B, Murty VP (2021) Increased functional coupling between VTA and hippocampus during rest in first-episode psychosis. eNeuro 8:1–8. 10.1523/ENEURO.0375-20.2021 33658310 PMC7986546

[B27] Griffa A, Baumann PS, Klauser P, Mullier E, Cleusix M, Jenni R, van den Heuvel MP, Do KQ, Conus P, Hagmann P (2019) Brain connectivity alterations in early psychosis: from clinical to neuroimaging staging. Transl Psychiatry 9:1–10. 10.1038/s41398-019-0392-y 30718455 PMC6362225

[B28] Hardy KV, Association AP, Ballon JS, Noordsy DL, Adelsheim S (2019) Intervening early in psychosis: a team approach first edit.

[B29] Howes OD, Williams M, Ibrahim K, Leung G, Egerton A, McGuire PK, Turkheimer F (2013) Midbrain dopamine function in schizophrenia and depression: a post-mortem and positron emission tomographic imaging study. Brain 136:3242–3251. 10.1093/brain/awt264 24097339 PMC3808688

[B30] Jalbrzikowski M, et al. (2021) Association of structural magnetic resonance imaging measures with psychosis onset in individuals at clinical high risk for developing psychosis: an ENIGMA working group mega-analysis. JAMA Psychiatry 78:753–766. 10.1001/jamapsychiatry.2021.0638 33950164 PMC8100913

[B31] Ji JL, et al. (2021) Mapping brain-behavior space relationships along the psychosis spectrum. Elife 10:1–83. 10.7554/eLife.66968 34313219 PMC8315806

[B32] Jiang Y, et al. (2023) Neuroimaging biomarkers define neurophysiological subtypes with distinct trajectories in schizophrenia. Nature Mental Health 1:186–199. 10.1038/s44220-023-00024-0

[B33] Kane JM, et al. (2016) Comprehensive versus usual community care for first-episode psychosis: 2-year outcomes from the NIMH RAISE early treatment program. Am J Psychiatry 173:362–372. 10.1176/appi.ajp.2015.15050632 26481174 PMC4981493

[B34] Kay SR, Fiszbein A, Opler LA (1987) The positive and negative syndrome scale (PANSS) for schizophrenia. Schizophr Bull 13:261–276. 10.1093/schbul/13.2.261 3616518

[B35] Kay SR, Opler LA, Lindenmayer JP (1988) Reliability and validity of the positive and negative syndrome scale for schizophrenics. Psychiatry Res 23:99–110. 10.1016/0165-1781(88)90038-8 3363019

[B36] Keshavan MS, Amirsadri A (2007) Early intervention in schizophrenia: current and future perspectives. Curr Psychiatry Rep 9:325–328. 10.1007/s11920-007-0040-8 17880865

[B37] Keshavan MS, DeLisi LE, Seidman LJ (2011) Early and broadly defined psychosis risk mental states. Schizophr Res 126:1–10. 10.1016/j.schres.2010.10.006 21123033 PMC3388534

[B38] Klein A, Tourville J (2012) 101 labeled brain images and a consistent human cortical labeling protocol. Front Neurosci 6:1–12. 10.3389/fnins.2012.00171 23227001 PMC3514540

[B39] Kolenič M, Španiel F, Hlinka J, Matějka M, Knytl P, Šebela A, Renka J, Hajek T (2020) Higher body-mass index and lower gray matter volumes in first episode of psychosis. Front Psychiatry 11:1–10. 10.3389/fpsyt.2020.556759 33173508 PMC7538831

[B40] Kring AM, Gur RE, Blanchard JJ, Horan WP, Reise SP (2013) The clinical assessment interview for negative symptoms (CAINS): final development and validation. Am J Psychiatry 170:165–172. 10.1176/appi.ajp.2012.12010109 23377637 PMC3785242

[B41] Lewandowski K, Bouix S, Ongur D, Shenton M (2020) Neuroprogression across the early course of psychosis. J Psychiatr Brain Sci 5:1–18. 10.20900/jpbs.20200002 32258424 PMC7111514

[B42] Lisman JE, Coyle JT, Green RW, Javitt DC, Benes FM, Heckers S, Grace AA (2008) Circuit-based framework for understanding neurotransmitter and risk gene interactions in schizophrenia. Trends Neurosci 31:234–242. 10.1016/j.tins.2008.02.005 18395805 PMC2680493

[B43] Lodge DJ, Grace AA (2011) Hippocampal dysregulation of dopamine system function and the pathophysiology of schizophrenia. Trends Pharmacol Sci 32:507–513. 10.1016/j.tips.2011.05.001 21700346 PMC3159688

[B44] Mahdiar M, Mohammadzade N, Homayooni A, Akhoundi FH, Kashaninasab F, Zamani B, Shariat SV, Shalbafan M, Rohani M (2023) Raphe nuclei echogenicity and diameter of third ventricle in schizophrenia measured by transcranial sonography. Basic Clin Neurosci 14:463–470. 10.32598/bcn.2021.1604.1 38050567 PMC10693814

[B45] Manera AL, Dadar M, Collins DL, Ducharme S (2019) Deformation based morphometry study of longitudinal MRI changes in behavioral variant frontotemporal dementia. Neuroimage Clin 24:102079. 10.1016/j.nicl.2019.102079 31795051 PMC6879994

[B46] Marcus DS, Fotenos AF, Csernansky JG, Morris JC, Buckner RL (2010) Open access series of imaging studies (OASIS): longitudinal MRI data in nondemented and demented older adults. J Cogn Neurosci 22:2677. 10.1162/jocn.2009.21407 19929323 PMC2895005

[B47] McHugo M, Avery S, Armstrong K, Rogers BP, Vandekar SN, Woodward ND, Blackford JU, Heckers S (2021) Anterior hippocampal dysfunction in early psychosis: a 2-year follow-up study. Psychol Med 53:160–169. 10.1017/S0033291721001318 33875028 PMC8919704

[B48] McHugo M, Roeske MJ, Vandekar SN, Armstrong K, Avery SN, Heckers S (2024) Smaller anterior hippocampal subfields in the early stage of psychosis. Transl Psychiatry 14. 10.1038/s41398-023-02719-5 38296964 PMC10830481

[B49] Mirzakhanian H, Singh F, Cadenhead KS (2014) Biomarkers in psychosis: an approach to early identification and individualized treatment. Biomark Med 8:51. 10.2217/bmm.13.134 24325224 PMC6984197

[B50] Moghaddam HS, Parsaei M, Taghavizanjani F, Cattarinussi G, Aarabi MH, Sambataro F (2024) White matter alterations in affective and non-affective early psychosis: a diffusion MRI study. J Affect Disord 351:615–623. 10.1016/j.jad.2024.01.238 38290585

[B51] O'Neill A, Mechelli A, Bhattacharyya S (2019) Dysconnectivity of large-scale functional networks in early psychosis: a meta-analysis. Schizophr Bull 45:579. 10.1093/schbul/sby094 29982729 PMC6483589

[B52] Paladini CA, Williams JT (2004) Noradrenergic inhibition of midbrain dopamine neurons. J Neurosci 24:4568. 10.1523/JNEUROSCI.5735-03.2004 15140928 PMC6729397

[B53] Purves-Tyson TD, Weber-Stadlbauer U, Richetto J, Rothmond DA, Labouesse MA, Polesel M, Robinson K, Shannon Weickert C, Meyer U (2019) Increased levels of midbrain immune-related transcripts in schizophrenia and in murine offspring after maternal immune activation. Mol Psychiatry 26:849–863. 10.1038/s41380-019-0434-0 31168068 PMC7910216

[B54] Rapado-Castro M, et al. (2021) Fronto-parietal gray matter volume loss is associated with decreased working memory performance in adolescents with a first episode of psychosis. J Clin Med 10:1–10. 10.3390/jcm10173929 34501377 PMC8432087

[B55] Romaniuk L, et al. (2010) Midbrain activation during pavlovian conditioning and delusional symptoms in schizophrenia. Arch Gen Psychiatry 67:1246–1254. 10.1001/archgenpsychiatry.2010.169 21135324

[B56] Rubinov M, Bullmore E (2013) Schizophrenia and abnormal brain network hubs. Dialogues Clin Neurosci 15:339. 10.31887/DCNS.2013.15.3/mrubinov 24174905 PMC3811105

[B57] Sato Y, Sakuma A, Ohmuro N, Katsura M, Abe K, Tomimoto K, Iizuka K, Ito F, Tomita H, Matsumoto K (2021) Relationship between white matter microstructure and hallucination severity in the early stages of psychosis: a diffusion tensor imaging study. Schizophr Bull Open 2:1–11. 10.1093/schizbullopen/sgab015

[B58] Schobel SA, et al. (2013) Imaging patients with psychosis and a mouse model establishes a spreading pattern of hippocampal dysfunction and implicates glutamate as a driver. Neuron 78:81–93. 10.1016/j.neuron.2013.02.011 23583108 PMC3966570

[B59] Shinn AK, et al. (2017) Mclean on track: a transdiagnostic program for early intervention in first-episode psychosis. Early Interv Psychiatry 11:83–90. 10.1111/eip.12299 26616380 PMC4884661

[B60] Si S, et al. (2024) Mapping gray and white matter volume abnormalities in early-onset psychosis: an ENIGMA multicenter voxel-based morphometry study. Mol Psychiatry 29:496–504. 10.1038/s41380-023-02343-1 38195979 PMC11116097

[B61] Smith SM, et al. (2006) Tract-based spatial statistics: voxelwise analysis of multi-subject diffusion data. Neuroimage 31:1487–1505. 10.1016/j.neuroimage.2006.02.024 16624579

[B62] Steinmann S, et al. (2021) Sex-related differences in white matter asymmetry and Its implications for verbal working memory in psychosis high-risk state. Front Psychiatry 12:1–10. 10.3389/fpsyt.2021.686967 34194350 PMC8236502

[B63] Straub KT, Hua JPY, Karcher NR, Kerns JG (2020) Psychosis risk is associated with decreased white matter integrity in limbic network corticostriatal tracts. Psychiatry Res Neuroimaging 301:1–20. 10.1016/j.pscychresns.2020.111089 32442837 PMC7293570

[B64] Tomyshev AS, Lebedeva IS, Akhadov TA, Omelchenko MA, Rumyantsev AO, Kaleda VG (2019) Alterations in white matter microstructure and cortical thickness in individuals at ultra-high risk of psychosis: a multimodal tractography and surface-based morphometry study. Psychiatry Res Neuroimaging 289:26–36. 10.1016/j.pscychresns.2019.05.002 31132567

[B65] Torres US, et al. (2016) Patterns of regional gray matter loss at different stages of schizophrenia: a multisite, cross-sectional VBM study in first-episode and chronic illness. Neuroimage Clin 12:1–15. 10.1016/j.nicl.2016.06.002 27354958 PMC4910144

[B66] Tritsch NX, Oh WJ, Gu C, Sabatini BL (2014) Midbrain dopamine neurons sustain inhibitory transmission using plasma membrane uptake of GABA, not synthesis. Elife 3:e01936. 10.7554/eLife.01936 24843012 PMC4001323

[B67] Xiao Y, Sun H, Shi S, Jiang D, Tao B, Zhao Y, Zhang W, Gong Q, Sweeney JA, Lui S (2018) White matter abnormalities in never-treated patients with long-term schizophrenia. Am J Psychiatry 175:1129–1136. 10.1176/appi.ajp.2018.17121402 30068259

[B68] Yang K, et al. (2022) A multimodal study of a first episode psychosis cohort: potential markers of antipsychotic treatment resistance. Mol Psychiatry 27:1184–1191. 10.1038/s41380-021-01331-7 34642460 PMC9001745

[B69] Zhang H, Schneider T, Wheeler-Kingshott CA, Alexander DC (2012) NODDI: practical in vivo neurite orientation dispersion and density imaging of the human brain. Neuroimage 61:1000–1016. 10.1016/j.neuroimage.2012.03.072 22484410

